# Strawberry and Achenes Hydroalcoholic Extracts and Their Digested Fractions Efficiently Counteract the AAPH-Induced Oxidative Damage in HepG2 Cells

**DOI:** 10.3390/ijms19082180

**Published:** 2018-07-26

**Authors:** María Teresa Ariza, Tamara Y. Forbes-Hernández, Patricia Reboredo-Rodríguez, Sadia Afrin, Massimiliano Gasparrini, Lucía Cervantes, Carmen Soria, Elsa Martínez-Ferri, Maurizio Battino, Francesca Giampieri

**Affiliations:** 1 Instituto Andaluz de Investigación y Formación Agraria y Pesquera (IFAPA), Consejería de Agricultura, Pesca y Desarrollo Rural, Junta de Andalucía, IFAPA de Churriana, Cortijo de la Cruz s/n, Churriana, 29140 Málaga, Spain; mariat.ariza@juntadeandalucia.es (M.T.A.); lucia.cervantes@juntadeandalucia.es (L.C.); maria.soria@juntadeandalucia.es (C.S.); elsa.martinez@juntadeandalucia.es (E.M.-F.); 2 Dipartimento di Scienze Cliniche Specialistiche ed Odontostomatologiche (DISCO)-Sez. Biochimica, Facoltà di Medicina, Università Politecnica delle Marche, 60131 Ancona, Italy; tamara.forbe@gmail.com (T.Y.F.-H.); preboredo@uvigo.es (P.R.-R.); dolla.bihs@gmail.com (S.A.); m.gasparrini@univpm.it (M.G.)

**Keywords:** strawberry, achenes, in vitro digestion, reactive oxygen species, apoptosis, AAPH

## Abstract

Strawberry fruits are highly appreciated by consumers worldwide due to their bright red color, typical aroma, and juicy texture. While the biological activity of the complete fruit has been widely studied, the potential beneficial effects of the achenes (commonly named seeds) remain unknown. In addition, when raw fruit and achenes are consumed, the digestion process could alter the release and absorption of their phytochemical compounds, compromising their bioactivity. In the present work, we evaluated the protective effects against oxidative damage of nondigested and digested extracts from strawberry fruit and achenes in human hepatocellular carcinoma (HepG2) cells. For that purpose, cells were treated with different concentration of the extracts prior to incubation with the stressor agent, AAPH (2,2′-azobis(2-amidinopropane) dihydrochloride). Subsequently, intracellular accumulation of reactive oxygen species (ROS) and the percentage of live, dead, and apoptotic cells were determined. Our results demonstrated that all the evaluated fractions were able to counteract the AAPH-induced damage, suggesting that the achenes also present biological activity. The positive effects of both the raw fruit and achenes were maintained after the in vitro digestion process.

## 1. Introduction

A common denominator in the occurrence of most chronic diseases is the presence of oxidative stress, defined as an imbalance between the production of radical species (mainly reactive oxygen species, ROS) and antioxidant defenses (AOx) [1,2].

There is solid scientific evidence that verifies the direct relationship between high fruit and vegetable consumption and the prevention of several degenerative diseases [3,4]. Dietary antioxidants such vitamins and phytochemicals—usually present in this sort of food—seem to play an important role in increasing endogenous antioxidant systems and therefore, in the preservation of the ROS/AOxs balance [5].

The strawberry (*Fragaria* × *ananassa*, Duch.) is among the most widely consumed fruits in the world [6] and is a natural source of health-promoting biomolecules [7] to which several biological activities, such as antioxidant, anti-inflammatory, antiatherogenic, and anticarcinogenic, have been attributed [8,9,10,11,12].

Botanically, the strawberry is an infructescence consisting of a fleshy floral receptacle that supports a cluster of real dry fruits (achenes) containing the seeds [13]. These two different tissues (flesh and achenes) significantly differ in terms of chemical composition and antioxidant capacity [14,15,16]. While the biological activity of the complete fruit or its isolated phenolic compounds has been widely studied, to the best of our knowledge, there are no published works that evaluate the beneficial properties of the achenes separately. Achenes are normally discarded during fruit transformation processes in the horticultural industry; however, they could represent a potential source of bioactive ingredients. 

In addition, it has been demonstrated that the health-promoting effects of food matrices depend not only on their chemical composition, but also on the release (bioaccessibility) and transformation of their bioactive molecules during the processes of digestion, absorption, and utilization by the organism (bioavailability) [17,18,19]. The bioaccessibility and bioavailability of individual phytochemicals notably differs; the most abundant compounds in the raw matrices are not necessarily those leading to the highest concentrations of active metabolites in target tissues [20].

In recent years, strawberry breeding programs have focused on the production of new varieties with a high yield and flavour, precocity, optimal fruit weight, and few misshapen fruits [21,22]. However, some innovative programs have started by using the content of functional compounds as the selection criteria [23], but this standard could be worthless if bioavailability and bioactivity are not taken into account. Indeed, when studying the role of bioactive compounds in human health, bioavailability is not always considered [24]. In that sense, no studies on the bioactivity of the digested fractions of strawberry fruit and achenes have been reported to date.

Thus, the aim of the present work was to evaluate the protective effects against oxidative damage of nondigested and digested extracts from the complete strawberry fruit and achenes in human hepatocellular carcinoma cells (HepG2) in order to determine the putative bioactivity from bioavailable extracts.

## 2. Results

The strawberry and achenes fractions used in the present study were previously characterized by our group [16]. Phenolic acids and hydrolysable tannins (36.65 ± 1.38 mg/100 g whole fruit), along with anthocyanins (33.27 ± 0.72 mg/100 g whole fruit), were the main families identified in the raw strawberry extract (RF), followed by flavanols (7.72 ± 0.29 mg/100 g whole fruit) and flavonols (6.49 ± 0.03 mg/100 g whole fruit). In the achenes hydroalcoholic extract (AC), the main phytochemical compounds were phenolic acids and hydrolysable tannins (2.97 ± 0.13 mg/100 g whole fruit), followed by flavonols (0.53 ± 0.02 mg/100 g whole fruit), flavanols (0.23 ± 0.01 mg/100 g whole fruit), and anthocyanins (0.21 ± 0.01 mg/100 g whole fruit). In contrast to RF, AC contained high proportions of hydrolysable tannins, but low quantities of anthocyanins, the main strawberry pigments. Regarding the phenolic composition of the fractions obtained after the in vitro digestion of strawberry and achenes, it was observed that in the fraction obtained after the gastric digestion of the complete fruits (GF), anthocyanins represented the major compounds (49.38 ± 1.62 mg/100 g whole fruit), while in the fraction obtained after the intestinal digestion (IF), this family of compounds was not identified and the phenolic acids and hydrolysable tannins (14.06 ± 0.85 mg/100 g whole fruit) were predominant. Similar results were observed for the fractions obtained after the gastric and intestinal digestion of the achenes (GAC and IAC, respectively).

### 2.1. Effects of Strawberry Fractions on Cell Viability

**Figure 1:**
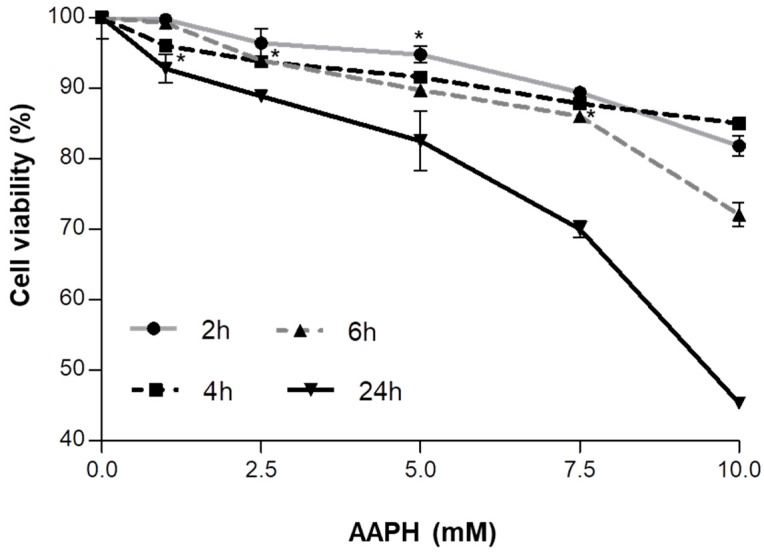
Viability of HepG2 determined by the MTT assay. Cells were incubated with different concentrations of AAPH (0–10 mM) at the indicated times. Values are expressed as the mean ± SD of three independent experiments (*n* = 3). * Indicates the concentrations from which significant differences (*p* < 0.05) were observed compared to the control.

**Figure 2:**
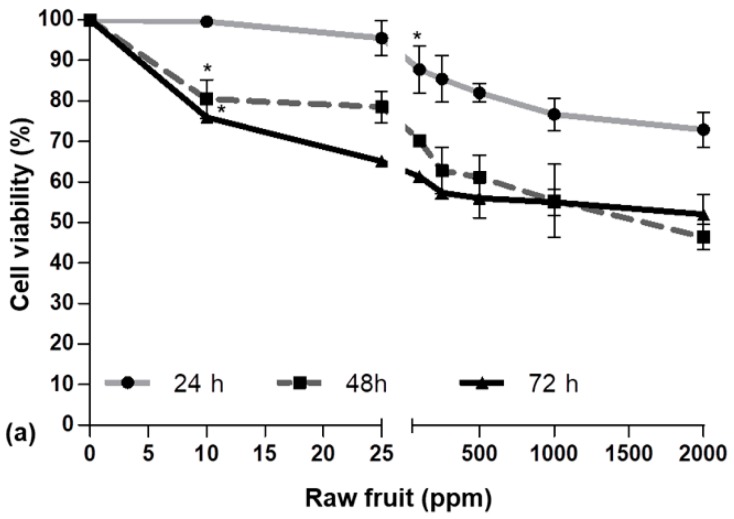

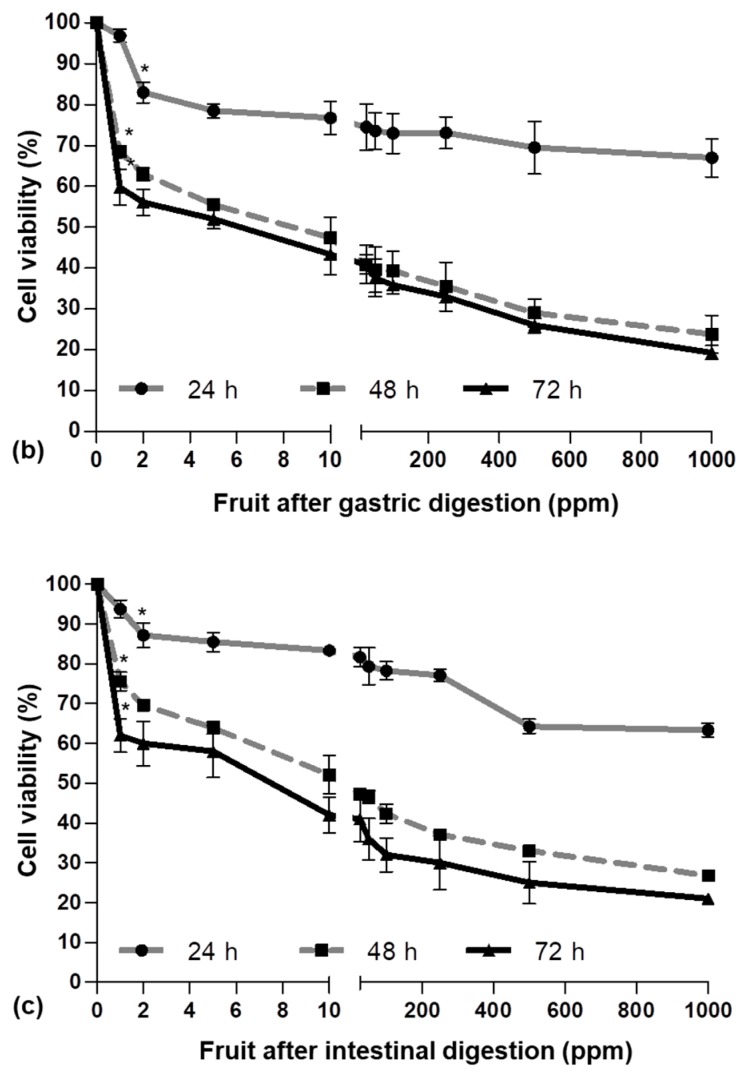
Viability of HepG2 determined by the MTT assay after incubation with different concentrations of (**a**) raw fruit; (**b**) fruit after gastric digestion; (**c**) fruit after intestinal digestion. Values are expressed as the mean ± SD of three independent experiments (*n* = 3). * Indicates the concentrations from which significant differences (*p* < 0.05) were observed compared to the control.

**Figure 3:**
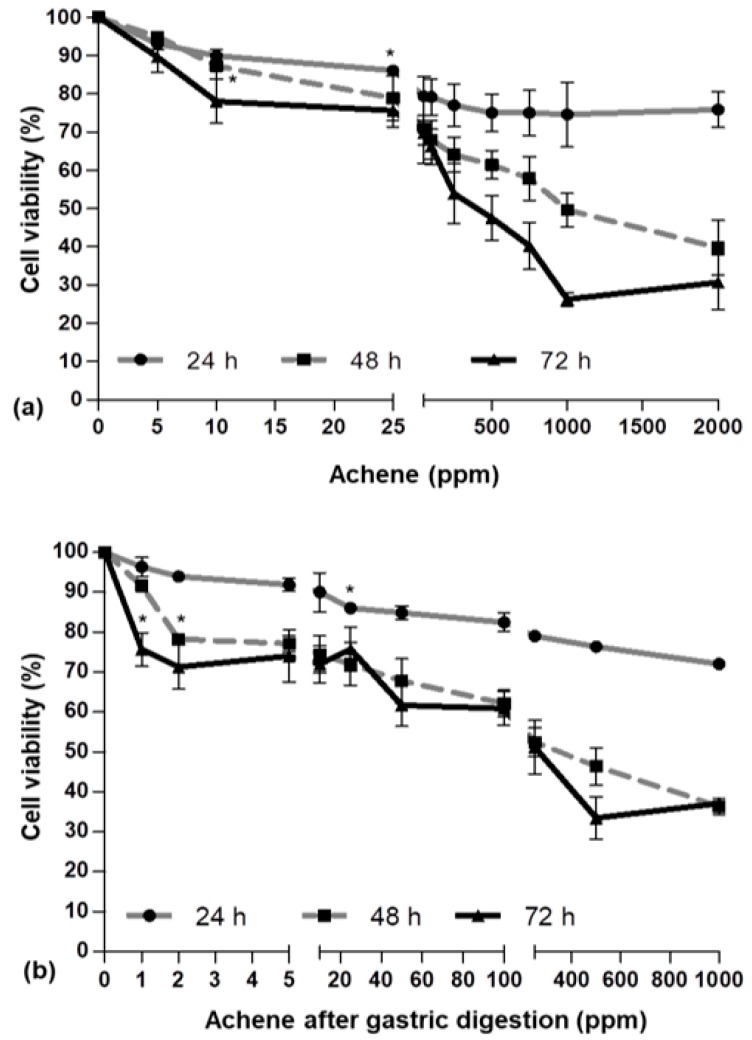

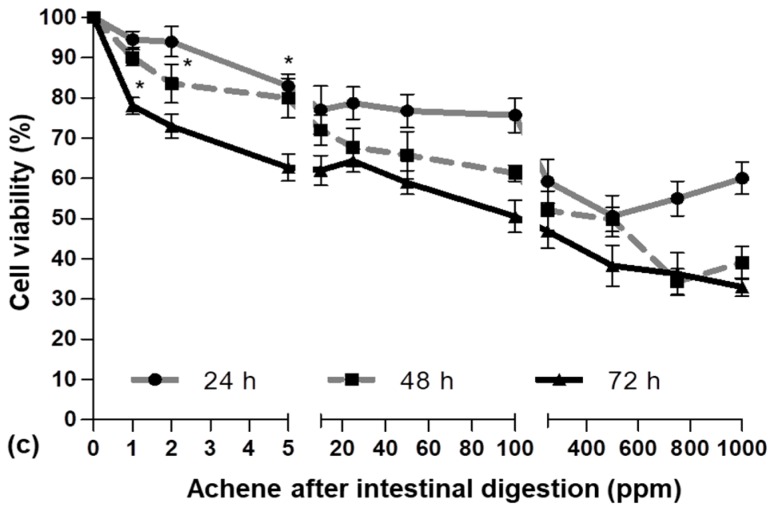
Viability of HepG2 determined by the MTT assay after incubation with different concentrations of (**a**) achene; (**b**) achene after gastric digestion; (**c**) achene after intestinal digestion. Values are expressed as the mean ± SD of three independent experiments (*n* = 3). * Indicates the concentrations from which significant differences (*p* < 0.05) were observed compared to the control.

In order to evaluate the AAPH (2,2′-azobis(2-amidinopropane) dihydrochloride) effect on cell viability and the potential cytotoxicity of the strawberry fruit and achene fractions, an MTT (3-(4,5-dimethylthiazol-2-yl)-2,5-diphenyltetrazolium bromide) assay was performed. Cells were incubated with (i) different concentrations of AAPH (0–10 mM) for 2, 4, 6, or 24 h; (ii) different concentrations of RF (0–2000 ppm), GF, or IF (0–1000 ppm) for 24, 48, or 72 h; and (iii) different concentrations of AC (0–2000 ppm), GAC, or IAC (0–1000 ppm) for 24, 48, or 72 h. As shown in Figure 1, Figure 2 and Figure 3, respectively, the effects on cell viability varied depending on the treatment’s concentration and the exposure time. 

After incubation with AAPH for 24 h, cell viability decreased from 100 to 45% (Figure 1), while RF caused a viability diminution from 100 to 73% (Figure 2a), GF from 100 to 67% (Figure 2b), and IF from 100% to 63% (Figure 2c). Likewise, AC, GAC, and IAC reduced cell viability by 24%, 28%, and 40%, respectively (Figure 3a–c). 

The concentrations of 25 µg/mL of RF; 10 µg/mL of AC; and 2 µg/mL of GF, IF, GAC, or IAC were used for subsequent experiments. For the stressor agent, the concentration selected was 2.5 mM. All these values guaranteed a cellular viability close to 90%.

### 2.2. Effects of Strawberry and Achenes Fractions on Intracellular ROS Production

**Figure 4:**
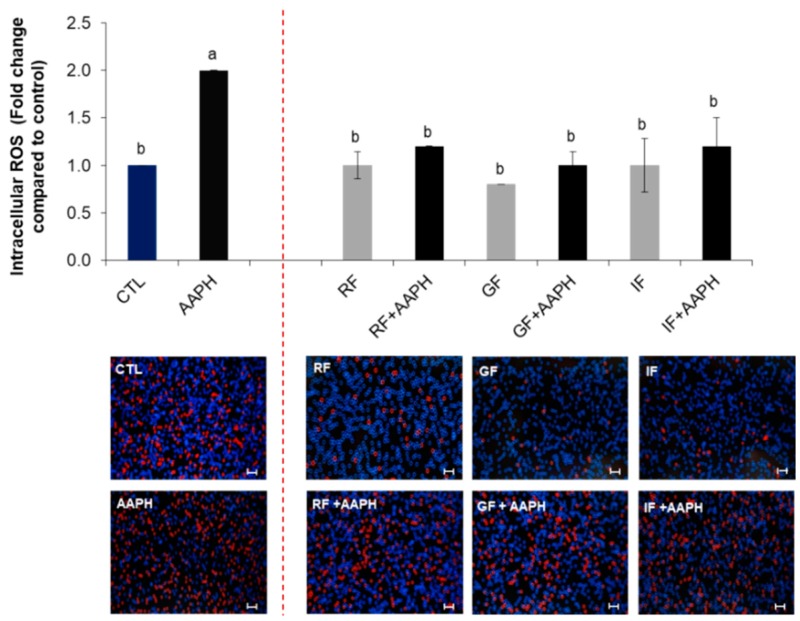
Intracellular reactive oxygen species (ROS) accumulation in HepG2 cells determined by the Tali^®^ Image-Based Cytometer. Cells were preincubated with the indicated fruit fractions and then stressed with AAPH for 24 h. Values are expressed as the mean ± SD of three independent experiments (*n* = 3). Columns belonging to the same set of data with different superscript letters are significantly different (*p* < 0.05). Representative images of intracellular ROS quantification are shown following the graphs (stressed cells appear red). Scale bar: 50 µm. CTL: cells without treatment; RF: cells incubated with the raw fruit; GF: cells incubated with fruit after gastric digestion; IF: cells incubated with fruit after intestinal digestion; AAPH: cells incubated with AAPH; RF + AAPH: cells preincubated with the raw fruit and then stressed with AAPH; GF + AAPH: cells preincubated with fruit after gastric digestion and then stressed with AAPH; IF + AAPH: cells preincubated with fruit after intestinal digestion and then stressed with AAPH.

**Figure 5:**
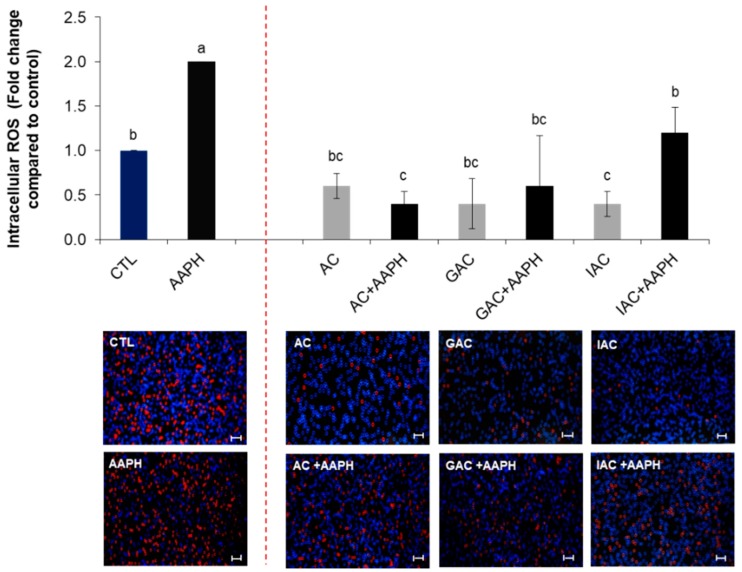
Intracellular reactive oxygen species (ROS) accumulation in HepG2 cells determined by the Tali^®^ Image-Based Cytometer. Cells were preincubated with the indicated achene fractions and then stressed with AAPH for 24 h. Values are expressed as the mean ± SD of three independent experiments (*n* = 3). Columns belonging to the same set of data with different superscript letters are significantly different (*p* < 0.05). Representative images of intracellular ROS quantification are shown following the graphs (stressed cells appear red). Scale bar: 50 µm. CTL: cells without treatment; AC: cells incubated with the achene; GAC: cells incubated with achene after gastric digestion; IAC: cells incubated with achene after intestinal digestion; AAPH: cells incubated with AAPH; AC + AAPH: cells preincubated with the achene and then stressed with AAPH; GAC+AAPH: cells preincubated with achene after gastric digestion and then stressed with AAPH; IAC + AAPH: cells preincubated with achene after intestinal digestion and then stressed with AAPH.

As shown in Figure 4 and Figure 5, treatment with AAPH increased the intracellular ROS levels by up to two times compared to control (untreated cells). Preincubation with any of the complete fruit fractions (RF, GF, IF) restored these values near to the baseline. However, when they were applied alone, they did not cause a ROS level reduction compared to untreated cells. Only GF caused a diminution under the control values (0.8-fold), but no significant (*p* < 0.05) differences were observed (Figure 4).

Regarding the achenes fractions, IAC resulted as being the most effective when applied without APPH (it significantly (*p* < 0.05) decreased intracellular ROS content by up to 0.4-fold compared to the control), while AC was the treatment that better counteracted the oxidative damage caused by the chemical stressor (Figure 5).

### 2.3. Effects of Strawberry and Achenes Fractions on Apoptosis

**Figure 6:**
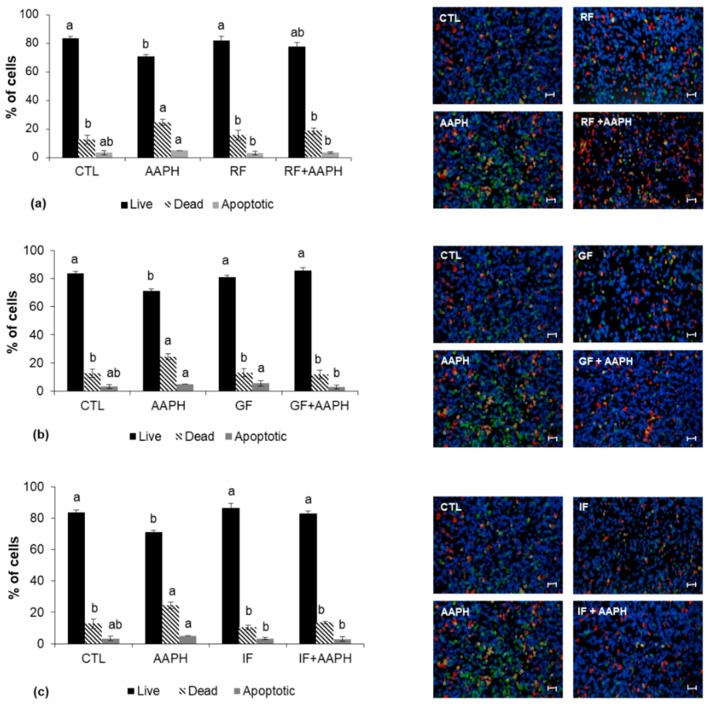
Percentage of live, dead, and apoptotic cells determined by the Tali^®^ Image-Based Cytometer. Cells were preincubated with (**a**) raw fruit; (**b**) fruit after gastric digestion; (**c**) fruit after intestinal digestion and then stressed with AAPH for 24 h. Values are expressed as the mean ± SD of three independent experiments (*n* = 3). Columns belonging to the same set of data with different superscript letters are significantly different (*p* < 0.05). Representative images of apoptosis quantification are shown following the graphs (stressed cells appear red). Scale bar: 50 µm. CTL: cells without treatment; RF: cells incubated with the raw fruit; GF: cells incubated with fruit after gastric digestion; IF: cells incubated with fruit after intestinal digestion; AAPH: cells incubated with AAPH; RF + AAPH: cells preincubated with the raw fruit and then stressed with AAPH; GF + AAPH: cells preincubated with fruit after gastric digestion and then stressed with AAPH; IF + AAPH: cells preincubated with fruit after intestinal digestion and then stressed with AAPH.

No significant differences (*p* < 0.05) were found in the percentage of live, dead, and apoptotic cells after treatment with the strawberry fractions (RF, GF, IF) compared to the control, whereas in the AAPH-stressed cells, the percentage of dead cells was significantly higher (*p* < 0.05; 1.93-fold). Pretreatment with any of the strawberry fractions prior to oxidative damage significantly reduced (*p* < 0.05) the number of dead and apoptotic cells with respect to the AAPH-stressed group and restored the percentage of live cells to values similar to the control. In that sense, the digested fractions (GF, IF) resulted as being more effective than the raw strawberry extract (RF) (Figure 6a–c).

**Figure 7:**
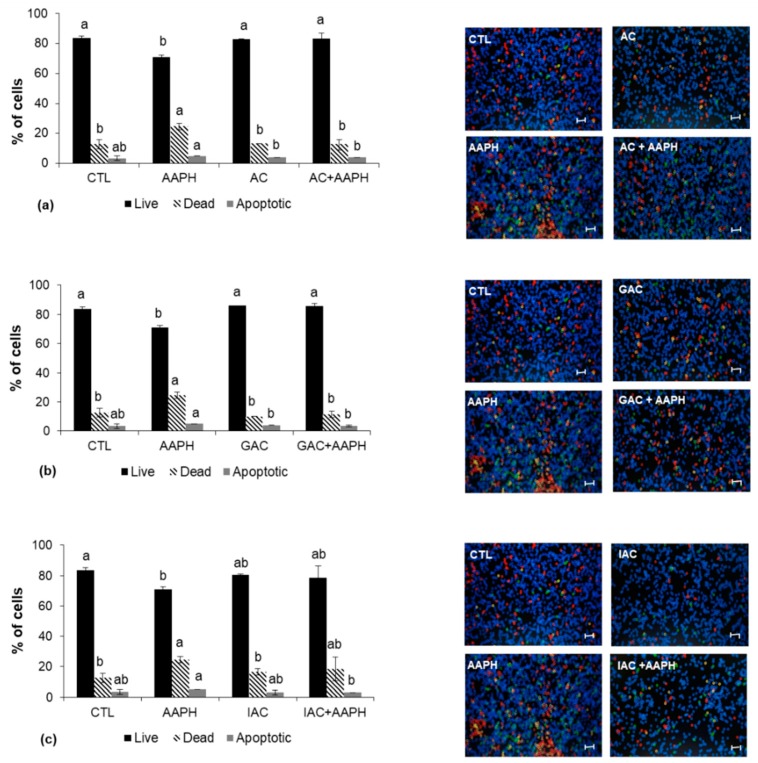
Percentage of live, dead, and apoptotic cells determined by the Tali^®^ Image-Based Cytometer. Cells were preincubated with (**a**) achene; (**b**) achene after gastric digestion; (**c**) achene after intestinal digestion and then stressed with AAPH for 24 h. Values are expressed as the mean ± SD of three independent experiments (*n* = 3). Columns belonging to the same set of data with different superscript letters are significantly different (*p* < 0.05). Representative images of apoptosis quantification are shown following the graphs (stressed cells appear red). Scale bar: 50 µm. CTL: cells without treatment; AC: cells incubated with the achene; GAC: cells incubated with achene after gastric digestion; IAC: cells incubated with achene after intestinal digestion; AAPH: cells incubated with AAPH; AC + AAPH: cells preincubated with the achene and then stressed with AAPH; GAC + AAPH: cells preincubated with achene after gastric digestion and then stressed with AAPH; IAC + AAPH: cells preincubated with achene after intestinal digestion and then stressed with AAPH.

Neither were significant differences (*p* < 0.05) found compared to the control in terms of live, dead, and apoptotic cells after the treatment with the different fractions of achenes (AC, GAC, IAC). Also in this case, AAPH treatment increased the number of apoptotic cells, while decreasing their vitality. Preincubation with any of the achenes fractions restored these values near to the baseline. AC and GAC were more effective in restoring cell vitality than the intestinal fraction, IAC (Figure 7a–c).

## 3. Discussion

In the last decades, oxidative stress has been recognized as one of the determining factors in the development and progression of most chronic noncommunicable diseases, including cardiovascular diseases, type 2 diabetes, and cancer [25,26].

The balance between the formation and elimination of the reactive nitrogen and oxygen species (the main free radicals generated in the organism) and consequently, the prevention of stress situations, is guaranteed by antioxidant defense systems, including enzymatic and nonenzymatic mechanisms. This last group refers, above all, to antioxidants of dietary origin, especially vitamins, minerals, and phytochemicals (polyphenols and carotenoids). From this derives the importance of diet as a factor involved in the modulation of oxidative stress [27,28,29,30].

In the present work, we demonstrated that strawberry and achenes hydroalcoholic extracts, as well as the fractions obtained after an in vitro digestion process, decreased the oxidative damage caused by a chemical agent. All the evaluated fractions (three of complete fruit: RF, GF, and IF; and three of achenes: AC, GAC, and IAC) were able to counteract the AAPH-induced damage as demonstrated by intracellular ROS accumulation and apoptosis data. Among the complete strawberry extracts, the digested fractions (GF, IF) counteracted to a greater extent the apoptosis and cell death caused by the AAPH with respect to RF, while no differences were observed among them in relation to the intracellular ROS production. In the case of the achenes fractions, the hydroalcoholic extract (AC) was the most effective in restoring the ROS values to baseline, and along with the fraction obtained after gastric digestion (GAC), showed the best results in terms of apoptosis inhibition.

Previous studies had already demonstrated that strawberry extracts improved the deleterious effects caused by different stressors such as AAPH [12], hydrogen peroxide (H_2_O_2_) [31], or lipopolysaccharide [32,33,34,35]; however, this is the first time that it is demonstrated that bioactivity is not lost during the digestion process. We also demonstrated for the first time that achene extracts have protective effects against oxidative damage.

Data describing the polyphenol concentration after in vivo digestion of strawberries are scarce. Azzini et al. [36] described that coumaric acid, protocatechuic acid, and hydroxbenzoic acid were the main compounds quantified in plasma after consuming fresh strawberries, while no traces of quercetin and kampferol were revealed. It is known that the bioavailability of the main phytochemicals of strawberries (anthocyanins) is relatively low (often less than 1% of the ingested dose appears in the plasma or is excreted in the urine) [37,38,39,40,41]; however, its contribution to health effects may be significant. Anthocyanins are absorbed mostly in their intact glycosidic or methylated forms, and are rapidly distributed into the circulatory system (within 0.25–2 h after ingestion) [38,40]. Their absorption mainly occurs in the stomach [42], while the environment of the small and large intestines (largely at neutral pH) do not favor their stability [16,38,39]. Furthermore, it has been suggested that a rapid degradation of these compounds within the gastrointestinal tract [38,40] and by the gut microbiota is likely to modify the anthocyanins’ molecular structures into phenolic acids, although modification by the small intestine conditions is more difficult to assess and remains yet to be described [38]. 

Regarding the achenes, there are no preceding works that have reported their digestion or metabolization. However, we previously identified and quantified several bioactive compounds in achenes extracts subjected to an in vitro digestion process, which suggested the successful release of these phytochemicals from this matrix [15,16].

Interestingly, the positive effects against the AAPH-induced stress were observed for all the fractions, independently of their chemical composition. In fact, the intestinal digested fractions (IF, IAC), where no anthocyanins were detected [16], also presented a positive biological activity. Generally, the protective effects of strawberry and many other berries have been attributed to this group of compounds [7,43,44]; however, our results indicated that other families, such as phenolic acids and hydrolysable tannins (mainly ellagic acid) or flavonols (such as myricetin, quercetin, and kaempferol), can also contribute to their bioactivity. In that sense, Chaudhuri et al. [45] and Álvarez et al. [46] reported that quercetin protected cellular membranes against oxidative damage and erythrocytes against cytosolic enzymatic depletion, lipid peroxidation, and haemolysis induced by AAPH. Likewise, ellagic acid protected human foetal lung (IMR-90) cells against oxidative DNA damage, suppressing the formation of 8-oxoguanine [47] and suppressed cytotoxicity and gluthathione loss induced by H_2_O_2_ and FeSO_4_ in neuronal PC12 cells [48]. The absorption of ellagic acid has been reported to occur rapidly after oral administration, with maximal blood concentration of 30–200 ng/mL and half-life of 8.4 h [49,50,51].

Our findings could be important for considering the achenes as a beneficial source of bioactive compounds and for incorporating this knowledge in order to select health-promoting varieties for breeding programs. Further studies are needed in order to confirm our findings, to explore the direct and indirect antioxidant mechanisms underlying the beneficial effects of strawberry and achenes digested fractions, and to further investigate the role of specific classes of compounds in explaining the reported bioactivities.

## 4. Materials and Methods

### 4.1. Preparation of the Strawberry and Achenes Extracts

Strawberries (*Fragaria* × *ananassa*, Duch, cv. *Camarosa*) were planted in mid-October 2015 at the experimental greenhouse of the Instituto Andaluz de Investigación y Formación Agraria y Pesquera (IFAPA) (Málaga, Spain), and seven months later three replications of 50 fresh and fully-ripened strawberry fruits were harvested. Half of the fruits were preserved, while the others were used to obtain the achenes. Both samples were lyophilized and stored at −20 °C until analysis. For the successive experiments, 6 extracts/fractions were obtained:

(i) Raw fruit (RF), corresponding to the hydroalcoholic extract of complete strawberries;
(ii) Fruit after gastric digestion (GF), corresponding to the fraction obtained after the gastric digestion of the complete fruits;
(iii) Fruit after intestinal digestion (IF), corresponding to the fraction obtained after the intestinal digestion of the complete fruits;
(iv) Achene (AC), corresponding to the hydroalcoholic extract of achenes;
(v) Achene after gastric digestion (GAC), corresponding to the fraction obtained after the gastric digestion of achenes;
(vi) Achene after intestinal digestion (IAC), corresponding to the fraction obtained after the intestinal digestion of achenes.

The hydroalcoholic extracts were obtained according to the procedure described by Ariza et al. [15], while the digested fractions were achieved following the in vitro digestion protocol described by Gil-Izquierdo et al. [52]. Samples were further concentrated under vacuum at 40 °C and stored at −80 °C until analysis. Next, the total phenolic, flavonoid, and anthocyanin content as well as the antioxidant capacity were determined. This antioxidant characterization was previously reported [15,16].

### 4.2. Culture of HepG2 Cells

HepG2 cells were purchased from the American Type Culture Collection (ATCC^®^ CL-173TM) and were grown in Dulbecco’s modified Eagle’s medium supplemented with 10% fetal bovine serum, 100 IU/mL penicillin, and 100 µg/mL streptomycin until reaching 80–90% confluence when subcultured. Cells were maintained in an HeraCell CO_2_ incubator at 37 °C with 5% CO_2_.

### 4.3. Determination of Cell Viability

For cell viability assessment, cells were seeded into 96-well plates at a density of 5 × 10^3^ cells/well, allowed to adhere for 16 h, and then treated with different concentrations (0, 1, 2, 5, 10, 25, 50, 100, 250, 500, 1000, and 2000 µg/mL) of the strawberry and achenes extracts (hydroalcoholic and digested fractions) for 24, 48, and 72 h. Cells were also incubated with different concentrations (0, 1, 2.5, 5, 7.5, and 10 mM) of the oxidative agent AAPH for 2, 4, 6, and 24 h. After incubation, 30 µL of RPMI medium containing 2 mg/mL of MTT was added to each well. Then, cells were incubated for 2 h at 37 °C in a 5% CO_2_ incubator. MTT solution was then discarded and 100 µL of dimethyl sulfoxide was added into each well to dissolve the formazan crystal. The level of colored formazan derivative was analyzed on a microplate reader (Thermo Scientific Multiskan EX, Monza, Italy) at a wavelength of 590 nm [53,54]. The percentage of cell viability was calculated according to the following equation:

% cell viability = (treated cells Abs/control cells Abs) × 100

where Abs: absorbance.

Data were reported as a mean value of three independent analyses ± standard deviation (SD).

### 4.4. Evaluation of Intracellular ROS Production

Evaluation of intracellular ROS production was performed through the CellROX^®^ Oxidative Stress Kit (Invitrogen TM, Life Technologies, Milan, Italy), according to the manufacturer’s instructions. Briefly, cells were seeded in 6-well plates at a density of 1.5 × 10^5^ cells/well and treated for 24 h with 25 µg/mL RF; 10 µg/mL AC; or 2 µg/mL IF, GF, GAC, or IAC in the presence or absence of 2.5 mM of AAPH. The concentrations employed for each fraction as well as the AAPH concentration were chosen according to the MTT viability assay, ensuring a vitality greater than 90%. After treatment, cells were washed twice with phosphate buffered saline (PBS) and detached by trypsinization with 0.5 mL of trypsin– ethylenediaminetetraacetic acid (EDTA) solution for 2–5 min at 37 °C in a 5% CO_2_ incubator. The trypsin was neutralized with 1.5 mL of complete medium, and cells were collected prior to centrifugation at 1500 rpm for 10 min at 4 °C. Then, the supernatant was discarded and the pellet was resuspended in 1 mL of complete medium. CellROX^®^ Orange Reagent was added at a final concentration of 5 µM, and samples were incubated for 30 min at 37 °C, centrifuged to remove medium and dye excesses, and resuspended again in PBS. After that, cells were analyzed with the Tali^®^ Image-Based cytometer (Thermo Fisher Scientific, Milan, Italy). Untreated cells, which were also labeled with CellROX^®^ Orange Reagent, were used to determine baseline levels of oxidative activity. Results were expressed as the fold increase of the intracellular ROS content compared to the control. Data were reported as a mean value of three independent analyses ± SD.

### 4.5. Apoptosis Quantification

For apoptosis quantification, the Tali™ Apoptosis Assay Kit–Annexin V Alexa Fluor^®^ 488 (Invitrogen TM, Life Technologies, Monza, Italy) was used according to the manufacturer’s instructions. Briefly, cells were seeded in 6-well plates at a density of 1.5 × 10^5^ cells/well and treated for 24 h with 25 µg/mL RF; 10 µg/mL AC; or 2 µg/mL IF, GF, GAC, or IAC in the presence or absence of 2.5 mM of AAPH. The concentrations employed for each fraction as well as the AAPH concentration were chosen according to the MTT viability assay, ensuring a vitality greater than 90%. After treatment, cells were washed twice with PBS and detached by trypsinization with 0.5 mL of trypsin–EDTA solution for 2–5 min at 37 °C in a 5% CO_2_ incubator. The trypsin was neutralized with 1.5 mL of complete medium and cells were collected prior to centrifugation at 1500 rpm for 10 min at 4 °C. Then, the supernatant was discarded and the pellet was resuspended in 100 mL of annexin binding buffer (ABB), and 5 mL of Annexin V Alexa Fluor^®^ 488 was added, mixed well, and incubated in the dark at room temperature for 20 min. Cells were then centrifuged at 1000 rpm for 5 min, resuspended in 100 mL of ABB, and then 1 mL of Tali™ Propidium Iodide was added, mixed well, and incubated in the dark at room temperature for 5 min. Samples were analyzed with the Tali^®^ Image-Based cytometer and the percentage of live, dead, and apoptotic cells was determined on the basis of the respective fluorescence. Data were reported as a mean value of three independent analyses ± SD.

### 4.6. Statistical Analysis

Statistical analyses were performed using Statistix software 9.0 (Analytical Software, Tallahassee, FL, USA). Results were subjected to ANOVA, and differences among means were located using Tukey’s honest significant difference (HSD) test.

## Abbreviations

ROS	Reactive oxygen species
AOx	Antioxidant defenses
HepG2	Human hepatocellular carcinoma cells
RF	Raw strawberry extract
AC	Achenes hydroalcoholic extract
GF	Fraction obtained after the gastric digestion of the complete fruits
IF	Fraction obtained after the intestinal digestion of the complete fruits
GAC	Fraction obtained after the gastric digestion of the achenes
IAC	Fraction obtained after the intestinal digestion of the achenes
AAPH	2,2′-Azobis(2-amidinopropane) dihydrochloride
MTT	3-(4,5-Dimethylthiazol-2-yl)-2,5-diphenyltetrazolium bromide
H_2_O_2_	Hydrogen peroxide
SD	Standard deviation
ABB	Annexin binding buffer
